# Sequencing and analysis of the complete mitochondrial genome of *Habrobracon hebetor* (Hymenoptera: Braconidae)

**DOI:** 10.1080/23802359.2020.1721026

**Published:** 2020-02-03

**Authors:** Yi-Xin Huang, Li-Qing Qi, Yan-Zhou Zhang, Xiang-Xiang Jin, Xu Wang

**Affiliations:** a Anhui Provincial Key Laboratory of the Conservation and Exploitation of Biological Resources, Key Laboratory of Biotic Environment and Ecological Safety in Anhui Province, College of Life Sciences, Anhui Normal University, Wuhu, Anhui, China;; b Key Laboratory of Zoological Systematics and Evolution, Institute of Zoology, Chinese Academy of Sciences, Beijing, China;; c Guangdong Key Laboratory of Animal Conservation and Resource Utilization, Guangdong Public Laboratory of Wild Animal Conservation and Utilization, Guangdong Institute of Applied Biological Resources, Guangzhou, Guangdong, China

**Keywords:** Ichneumonoidea, mtDNA, phylogenetic relationship

## Abstract

We determined the complete mitochondrial genome sequence of *Habrobracon hebetor* (Say). The complete mitogenome sequence of *H. hebetor* was observed to be a circular molecule 15,708 bp long and consists of 13 protein-coding genes (PCG), 2 ribosomal RNA (rRNA) genes, and 22 transfer RNA (tRNA) genes (GenBank accession no. MN842279). This nucleotide composition is biased toward adenine and thymine (85.2% A + T). The A + T-rich region is found between *trnM* and *trnQ*, and this entire region was 864 bp long.

The gregarious parasitoid *Habrobracon hebetor* (Say) (Hymenoptera: Braconidae) is a natural enemy of late larval stages of several field and stored-product lepidopterous pests, which has been studied as a control agent of various lepidopteran pests in China (Huang 1986). However, the mitogenome sequence of *H. hebetor* remains unknown so far. Here, we sequenced the complete mitochondrial DNA genome of *H. hebetor* to provide more comprehensive data toward establishing its relationship within the family Braconidae.

Adult *H. hebetor* were collected from Hefei City (N31°51′43.87′′ and E117°15′19.19′′), Anhui, China in August of 2019 and deposited in the Entomological Museum, College of Life Sciences, Anhui Normal University (AHNU) under the accession no. AHHF20190058.

The complete mitochondrial genome of *H. hebetor* was sequenced using an Illumina HiSeq2000 system made by the Shanghai Personal Biotechnology Limited Company (Shanghai, China). The annotation was carried out in Geneious 8.1.3 (Kearse et al. 2012). Protein-coding genes (PCG) were determined by the open reading frames; rRNAs and tRNAs were identified using MITOS (Bernt et al. 2013).

The *H. hebetor* mitochondrial genome is 15,708 bp (GenBank accession no. MN842279) in length with a total A + T content of 85.2% that is heavily biased toward the A and T nucleotides. It encodes the complete set of 37 genes which are usually found in animal mitogenomes. In the mitogenome of *H. hebetor*, a total of 33 bp overlaps have been found at 10 gene junctions. The mitogenome is loose and has a total of 150 bp intergenic sequences without the putative A + T-rich region. The intergenic sequences are at 17 locations ranging from 1 to 37 bp, with the longest one located between *atp6* and *cox3*. The A + T-rich region of the *H. hebetor* is 864 bp long and located between the *trnM* and *trnQ*.

All 22 tRNA genes usually found in the mitogenomes of insects are present in *H. hebetor*. The nucleotide length of tRNA genes ranges from 64 bp (*trnT*) to 71 bp (*trnK*), and A + T content ranges from 79.1% (*trnM*) to 95.6% (*trnW*). These two rRNA genes have been identified on the N-strand in the *H. hebetor* mitogenome.

We analyzed the nucleotide sequences of PCGs using the maximum-likelihood (ML) method to understand the phylogenetic relationship of *H. hebetor* with other Braconidae species. The mitogenome sequence of Ichneumonidae sp. was used as the outgroup. Our results show that *H. hebetor* belongs to the family Braconidae and is closely related to *Spathius agrili* (Figure 1).

**Figure 1.**
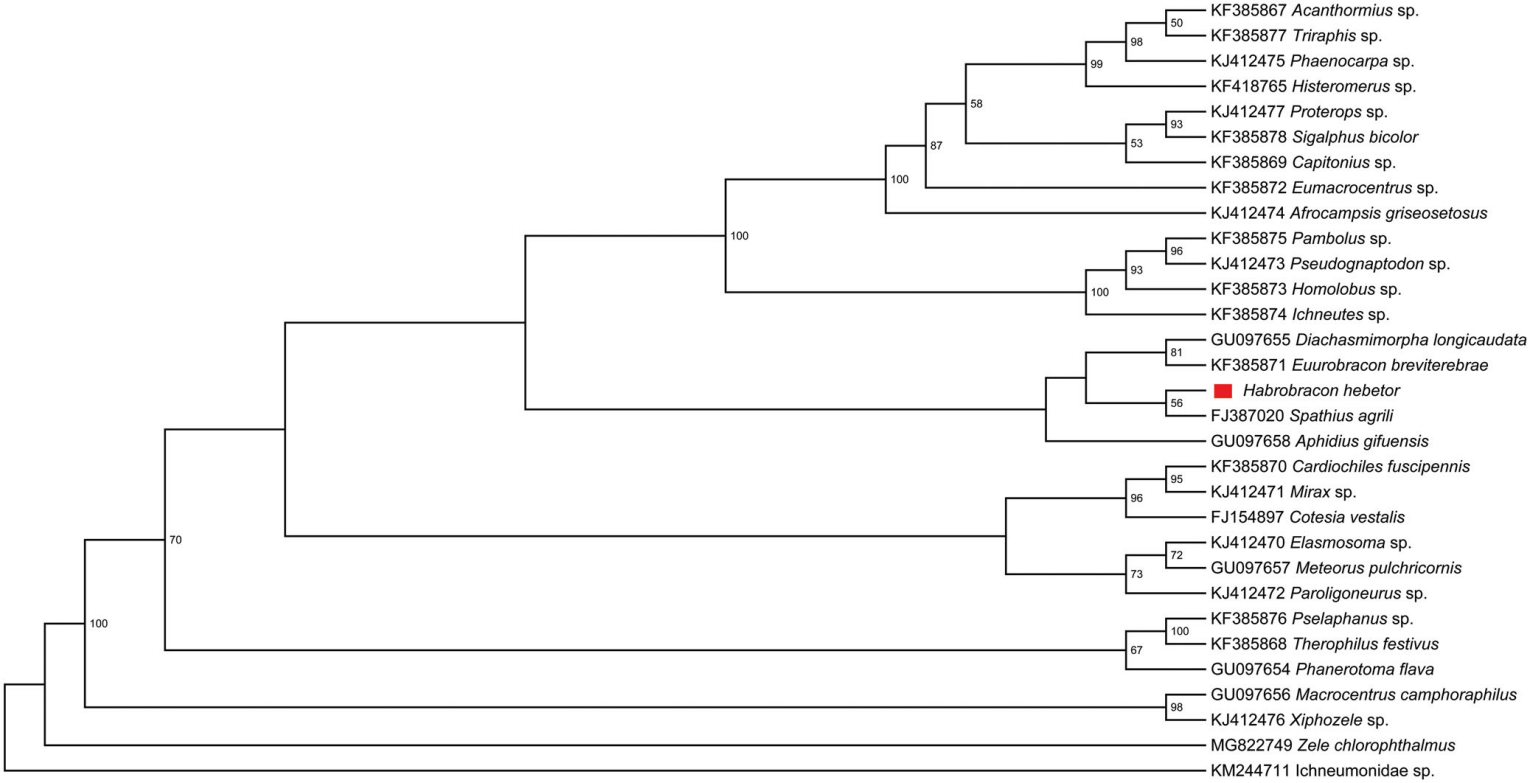
The maximum-likelihood (ML) phylogenetic tree of *Habrobracon hebetor* and other Braconidae species. The numbers beside the nodes are percentages of 1000 bootstrap values. Alphanumeric terms indicate the GenBank accession numbers.
